# A pilot Tuning Project-based national study on recently graduated medical students’ self-assessment of competences - the TEST study

**DOI:** 10.1186/s12909-015-0517-9

**Published:** 2015-12-19

**Authors:** Pedro Grilo Diogo, Joselina Barbosa, Maria Amélia Ferreira

**Affiliations:** Department of Medical Education and Simulation, Faculty of Medicine - University of Porto (FMUP), Porto, Portugal

**Keywords:** Student grouped self-assessments, Core competences, Tuning Project, Outcome-based program evaluation, Undergraduate medical education

## Abstract

**Background:**

The Tuning Project is an initiative funded by the European Commission that developed core competences for primary medical degrees in Europe. Students' grouped self-assessments are used for program evaluation and improvement of curricula. The TEST study aimed to assess how do Portuguese medical graduates self-assess their acquisition of core competences and experiences of contact with patients in core settings according to the Tuning framework.

**Methods:**

Translation of the Tuning's competences (Clinical Practice - CP), Knowledge (K) items and Clinical Settings (CS) was performed. Questionnaires were created in paper and electronic formats and distributed to 1591 graduates from seven Portuguese medical schools (July 2014). Items were rated in a 6-point Likert scale (0-5) of levels of competence. Exploratory factor analysis (EFA) was conducted and Cronbach's alpha was used to evaluate the internal consistency of the questionnaire. Kruskal-Wallis and Dunn's tests were used for multiple comparisons.

**Results:**

Three hundred eighty seven questionnaires were analyzed, corresponding to 24 % of the target population. EFA yielded an 11-factor solution for CP and a 6-factor solution for K items. The median value of CP factors was 2.8 (p25 = 2.0; p75 = 3.5) and the median value of K factors was 2.6 (2.0; 3.2). Factor scores ranged from 1.3 (Legal principles) to 4.0 (Ethical principles). Clinical presentations, psychological aspects of illness, evidence-based medicine and promotion of health showed the highest results. Lower scores were detected in medical emergencies, practical procedures, prescribing drugs and legal principles. More than 90 % of graduates experienced having contact with patients in 8 CS but only 24 % of graduates had contact in all 14 CS. Graduates had the least contact with patients in the emergency rooms, intensive care units, palliative, rehabilitation and anesthetic care. Significant differences (*p* < 0.05) among schools were detected in 8 factors and 7 settings.

**Conclusions:**

We developed a valid questionnaire supporting national SWOT analysis on the acquisition of core competences in medical education. Results suggest that Portuguese graduates are not fully prepared for clinical practice. Curricular improvements in core competences and the educational development of the transition period between undergraduate and postgraduate education ought to be considered. Outcome-based program evaluation relying on graduates’ grouped self-assessments contributes to inform changes in medical education.

## Background

The basis of outcome-based education (OBE) was put forward by Tyler [1] in 1949. Instead of the educational process, OBE looks upon education with an emphasis on predetermined learning outcomes, which superintend decisions about the curricula, teaching-learning strategies and the assessment of students [2]. From 2000 onwards, OBE gained wide support and took the lead as the mainstream paradigm in the education of healthcare professionals [3].

OBE has the advantage of providing a framework for quality assurance. Outcome-based program evaluations focus on the achievement of learning outcomes at the end of specific periods of education. If outcomes are not achieved, possible problems within curricula may be detected which drive the implementation of necessary improvements [2].

Medical schools and specialist organizations have developed several sets of learning outcomes, both for undergraduate and postgraduate education [4–9]. The Tuning Project [5] is an initiative funded by the European Commission which developed learning outcomes/competences for primary medical degrees in Europe. Tuning generated learning outcomes through an iterative process of expert review after an Europe-wide internet-based survey. Tuning’s core competences can assist curriculum planning or provide a framework for quality enhancement initiatives, given that they are designed to assure European standards of fitness for practice for medical graduates.

Several sources of data should be employed for a detailed analysis of medical curricula on an outcome-based paradigm. Student surveys are widely used by higher education institutions throughout the world for program evaluation purposes [10]. Although student self-assessments are acknowledged as inaccurate measures of competence at the individual level [11–13], research suggests that reliable grouped self-assessments are valid measures of the acquisition of learning outcomes by a group of students and thus important for program evaluation [14, 15]. In fact, student grouped self-assessments have guided curriculum planning [16], revisions of curricula [17, 18] and supported studies on graduates’ preparedness for practice doctors [19–22]. Also, the Greek medical schools have developed a self-assessment questionnaire based on Tuning’s core competences [23].

Nevertheless, examples of systematic outcome-based program evaluations based on student self-assessments against international sets of learning outcomes are scarce in the literature. In the Portuguese context, graduates’ self-assessments right after completion of the medical course may be particularly useful for program evaluation. In fact, there is no national summative assessment in Portugal for all medical graduates blueprinted against an agreed set of learning outcomes, although Portuguese medical schools have developed learning outcomes for graduates in Medicine [24]. The purpose of the national exam to access residency is to rank medical graduates instead of assessing their clinical competence. On the other hand, schools still struggle with the development of the comprehensive and valid assessment tools. Besides a comparative study of Portuguese-speaking countries by this research group [25], no other national study has been conducted on an outcome-based paradigm.

Recent changes have been introduced in medical education in Portugal, namely the attribution of full professional autonomy to all medical graduates who complete one year of internship (the ‘Ano Comum’ or ‘Common Year’), after which they enroll in specialty training. These changes in autonomy rules and foreseeable reforms in the Common Year and national exam highlight the need to produce research on the effectiveness of undergraduate programs in delivering core competences.

We designed the TEST study with the aim to assess whether Portuguese medical graduates perceive deficits in the acquisition of core competences and experienced of contact with patients in core clinical settings according to the Tuning framework, and whether there are significant differences among graduates from different medical schools.

## Methods

This cross-sectional study was based on a survey conducted between October and December 2014. The study population included all 1591 graduates who had recently finished their medical courses in July 2014 in seven Portuguese medical schools. Graduates answered the survey before access to residency, which started in January 2015.

### Study instrument

A questionnaire was created after translation of the “Tuning Project Learning Outcomes/Competences for Undergraduate Medical Education in Europe” [5].

Two researchers independently translated 12 Level 1 Tuning competences and their corresponding Level 2 competences related to Clinical Practice (70 items). Seven Knowledge domains (encompassing 39 items) and 14 Clinical Settings were also translated. Medical Professionalism outcomes were not included. A third researcher integrated both translations and created the final questionnaire, which was constituted by 123 items, including 70 Level 2 competences (*Clinical Practice*), 39 *Knowledge* items and 14 *Clinical Settings*.

Categorical demographic information was collected on participants' gender, age, medical school and modality of admission to the medical course (including General, Graduate and other contingents). Regarding *Clinical Practice* and *Knowledge* items*,* participants were asked to self-assess their level of competence or knowledge. A 6-point Likert scale from 0 to 5 was used (0 = non-existent, 1 = insufficient, 2 = sufficient, 3 = good, 4 = very good and 5 = excellent). Regarding *Clinical Settings,* participants were asked (yes or no question) if they had experienced contact with patients in specific learning settings (*Clinical Settings* were represented as CS*n*, being *n* its number).

### Implementation and sample size

Questionnaires were created in paper and electronic formats. Paper questionnaires were distributed by student volunteers to all graduates who attended preparation lectures for the national exam to access residency in the cities of Braga, Porto, Lisbon and Coimbra. 208 questionnaires were distributed and received. The electronic questionnaire was created in Google Forms and was diffused by e-mail through school-maintained mailing lists and through Facebook groups that are specific for medical graduates. 218 graduates filled the electronic questionnaire. Both versions of the questionnaire included an introduction to the purpose of the study. Explicit instructions for participants to fill only one of the versions of the questionnaire were given, in order to reduce the possibility of multiple responses per person. A total of 426 questionnaires were obtained.

We aimed for a sample size of five participants per item in the questionnaire (both for *Clinical Practice* and *Knowledge* components) in line with recommendations for the development of exploratory factor analysis (EFA) [26].

Questionnaires delivered back in blank or without full demographic information were excluded from analysis. We considered that the statistical analysis would likely be biased if responses to more than 10 % of items were missing [27].

This study was approved by the Faculty of Medicine of the University of Porto/São João Hospital Ethics Committee, in compliance with the Helsinki Declaration. Participation was voluntary and no incentives were offered. Completion of the questionnaire constituted consent to participate. Collected data were analyzed in an anonymous way and it was not possible for the researchers to identify the participants during any phase of the study.

### Statistical analysis

Data from paper questionnaires were read by a Fujitsu fi-5120c machine and entered a Microsoft Excel spreadsheet. Data from electronic questionnaires were downloaded from Google Forms into an Excel spreadsheet. Both data were fused in an Excel spreadsheet and transported to Statistical Package for Social Sciences (SPSS) version 21.0.

The relational structure of the *Clinical Practice* and the *Knowledge* items of the questionnaire was assessed by exploratory factor analysis (EFA). Factors were extracted after main components analysis followed by an oblique rotation. The factor structure was based on the scree plot criteria, eigenvalues and percentage of variance explained. To assess the adequacy of EFA, Kaiser-Meyer-Olkin (KMO) and Bartlett’s sphericity tests were conducted. An item was considered to belong to a certain factor when its factor load - with respect to that factor - was equal or greater than |0.35|, and the highest of the ones that fulfilled the previous condition. The internal consistency of the questionnaire was analyzed by Cronbach’s alpha (α).

Factor scores were described by the median value of its items and the 25th and 75th percentiles (p25; p75). Kruskal-Wallis and Dunn tests were used for multiple comparisons of factor scores and clinical settings among medical schools. Random numbers from 1 to 7 were attributed to medical schools for the purpose of multiple comparisons.

## Results

### Sample size and participants’ characteristics

Twenty seven percent of the study population answered the questionnaire and a sample size of 22 % or more graduates was obtained in all medical schools. Sample sizes ranged from 33 % (ECS-UM) to 22 % (FMUL and NMS/FCM) (Table 1). 387 paper and electronic questionnaires were analyzed, after implementation of exclusion criteria. 39 paper questionnaires were excluded: ICBAS (1), FMUC (2), University of Algarve (15) and foreign schools (21).

**Table 1:** Study population and sample sizes per medical school

Medical school	Study population^a^	Sample size (%)
ECS-UM	100	33 (33)
ICBAS	194	48 (25)
FMUP	242	70 (29)
FCS-UBI	145	33 (23)
FMUC	293	67 (23)
FMUL	371	81 (22)
NMS/FCM	246	55 (22)
Total	1591	387 (24)

^a^ Number of graduated students in July 2014. Information provided by the medical schools

Participants’ age ranged from 23 to 40 years old. 66.9 % (259) of the graduates were female and 33.1 % (128) were male. In the sample, the percentage of graduates from different medical schools ranged from 20.9 % (FMUL) to 8.5 % (ECS-UM and FCS-UBI), in accordance to each school’s number of admissions (Table 2). 85.8 % (332) of graduates were admitted to medical schools by the general contingent, while 6.5 % (25) were admitted by the graduate contingent and 7.0 % (27) by other contingents. Cohorts of graduates from different medical schools did not differ significantly in gender, age and modality of admission (*p* > 0.05, all three cases).

**Table 2:** Participants’ characteristics

Gender	N (%)
Female	259 (66.9)
Male	128 (33.1)
Medical school ^a^	
ECS-UM	33 (8.5)
ICBAS	48 (12.4)
FMUP	70 (18.1)
FCS-UBI	33 (8.5)
FMUC	67 (17.3)
FMUL	81 (20.9)
NMS/FCM	55 (14.2)
Admission contingent ^b^	
General contingent	332 (85.8)
Graduate contingent	25 (6.5)
Other contingents	27 (7.0)

^a^ The chi-square test showed no differences between graduates from different schools in terms of gender (chi-square = 11.410, *p* = 0.076) or admission contingent (chi-square = 17.733, *p* = 0.124)

^b^ Three participants omitted the admission modality

Missing responses were analyzed: since the maximum absence rate response was 3.9 %, the values of unanswered items were replaced by the median.

### Statistical analysis

Bartlett’s sphericity test was statistically significant (*p* < 0.0001) for both *Clinical Practice* and *Knowledge* items, indicating that items shared a common variance. The KMO test yielded 0.926 for *Clinical Practice* and 0.941 for *Knowledge* items, suggesting that they represent more than one factor.

EFA produced an 11-factor solution for *Clinical Practice* and a 6-factor solution for *Knowledge* items (Tables 3 and 4). *Clinical Practice* and *Knowledge* factors were labeled in accordance to Tuning’s descriptions. *Clinical Practice* factors were codified as CP*n* (being *n* its number) and *Knowledge* factors were codified as K*n*.

**Table 3:** Exploratory factor analysis results for Clinical Practice factors

Factors	Label	N^a^	Loadings (min, max)	Eigenvalue	Variance explained (%)	α
CP1	Clinical presentations and diagnosis	6	0.463, 0.646	1.8	2.7	0.851
CP2	Consultation and management plan	6	0.535, 0.707	2.4	3.7	0.868
CP3	Medical emergencies	6	0.502, 0.834	2.0	3.0	0.901
CP4	Prescribe drugs	3	0.779, 0.800	1.4	2.1	0.890
CP5	Practical procedures	11	0.350, 0.829	21.7	32.8	0.910
CP6	Communication in medical context	10	0.469, 0.650	2.5	3.7	0.908
CP7	Ethical principles	3	0.607, 0.652	1.2	1.8	0.771
CP8	Legal principles	3	0.627, 0.746	1.5	2.3	0.839
CP9	Psychological and social aspects of illness	3	0.697, 0.744	1.2	1.9	0.954
CP10	Evidence-based medicine, technology and science	8	0.401, 0.874	4.6	7.0	0.910
CP11	Population health and health care system	7	0.517, 0.721	3.7	5.6	0.904

^a^ Total number of items. Clinical Practice items number 22, 23 and 55 were removed since the internal reliability of the respective factors increased with their exclusion; item 35 was removed because of its low factor load (0.30)

**Table 4:** Exploratory factor analysis results for Knowledge factors

Factors	Label	N^a^	Loadings (min, max)	Eigenvalue	Variance explained (%)	α
K1	Basic and clinical sciences	11	0.577, 0.820	16.3	41.7	0.929
K2	Behavioural and social sciences	3	0.740, 0.807	1.4	3.6	0.892
K3	Drugs and prescribing	8	0.488, 0.806	2.2	5.5	0.928
K4	Public Health	10	0.442, 0.872	4.4	11.2	0.919
K5	Ethical principles	3	0.633, 0.735	1.3	3.3	0.919
K6	Role in healthcare systems	4	0.728, 0.793	2.6	6.6	0.942

^a^ Total number of items. No items were excluded

Eleven *Clinical Practice* and 6 *Knowledge* factors explained 66.6 % and 71.9 % of the total variance of answers, respectively. *Clinical Practice* and *Knowledge* factors showed a global α of 0.971 (min α = 0.771-max α = 0.954), and a global α of 0.965 (minα = 0.892-maxα = 0.942), respectively.

The aggregation of items into factors showed 4 differences in relation to Tuning’s groups of competences: 1) items 1 to 6 (under Level 1 ‘*Consultation with a patient’*) and items 7 to 12 (under Level 1 ‘*Clinical presentations, investigations, differential diagnoses and management plan*’) were aggregated into two factors (CP1 and CP2, which included, respectively, items 1, 2, 3, 7, 8 and 9, and items 4, 5, 6, 10, 11 and 12), 2) six items under Level 1 ‘*Ethical and Legal principles in medical practice*’ represented two separate factors (CP7-Ethical principles and CP8-Legal principles), 3) three Level 1 competences (‘*Principles, skills and knowledge of evidence-based medicine’*, ‘*Information and information technology effectively in a medical context’* and *‘Scientific principles*, *method and knowledge to medical practice’*) were combined in a single factor (CP10) and 4) two Knowledge domains (‘*Basic Sciences’* and ‘*Clinical Sciences’*) produced a single factor (K1).

### Factor scores

The median value of *Clinical Practice* (CP) factors was 2.8 (p25 = 2.0; p75 = 3.5) and the median value of *Knowledge* (K) factors was 2.6 (2.0; 3.2) in a scale from 0 to 5.

**Fig. 1:**
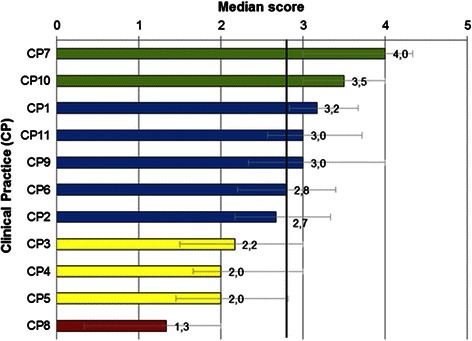
Clinical Practice factor scores. The error bars represent the interquartile range (p25-p75). Colors represent levels of competence: green bars (very good), blue (good), yellow (sufficient), red (insufficient). The vertical line corresponds to the median score of all Clinical Practice factors (2.8)

**Fig. 2:**
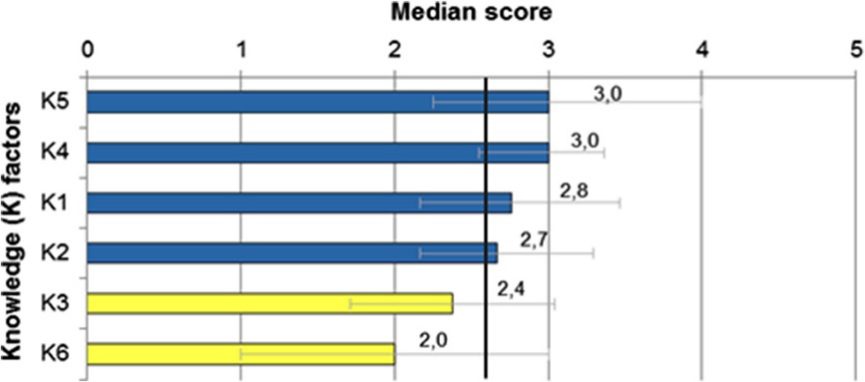
Knowledge factor scores. The error bars represent the interquartile range (p25-p75). Colored bars represent levels of competence: blue (good) and yellow (sufficient). The vertical line corresponds to the median score of all Knowledge factors (2.6)

CP factor scores ranged from 1.3 (CP8: Legal principles) to 4.0 (CP7: Ethical principles), which correspond, respectively, to insufficient and very good levels of competence. K factor scores ranged from 2.0 (K6: Role in health care systems) to 3.0 (K4: Public Health and K5-Ethical principles), corresponding to good levels of competence (Figs. 1 and 2).

Factors CP1, CP7, CP10, CP11, K1, K2, K4 and K5 scored above the respective CP or K median value while factors CP2, CP3, CP4, CP5, CP8, K3 and K6 scored below the median values.

CP6 (Communicate in medical context) equaled the CP median value. Results from the items aggregated in this factor ranged from 2.58 and 2.63 (“communicate with disabled people” and “communicate in breaking bad news”) to 4.00 (“communicating with patients” or “communicating with colleagues”).

Graduates self-assessed their competence as very good in CP7 and CP10. Good levels of competence were perceived in the majority of factors. Sufficient levels of competence were shown in CP3, CP4, CP5, K3 and K6. An insufficient level of competence was obtained in CP8 (Table 5).

**Table 5:** Levels of self-assessed competence in Clinical Practice and Knowledge factors

Factor and label	Median value^a^	Levels of competence
CP7 - Ethical principles	4.0	Very good (3.5 to 5.0)
CP10 - Evidence-based medicine, technology and science	3.5	
CP1 - Clinical presentations and diagnosis	3.2	Good (2.5 to <3.5)
CP9 - Psychological and social aspects of illness	3.0	
CP11 - Population health and health care system	3.0	
K4 - Public health	3.0	
K5 - Ethical principles	3.0	
CP6 - Communication in medical context	2.8	
K1 - Basic and clinical sciences	2.8	
CP2 - Consultation and management plan	2.7	
K2 - Behavioural and social sciences	2.7	
K3 - Drugs and prescribing	2.4	Sufficient (1.5 to <2.5)
CP3 - Medical emergencies	2.2	
K6 - Role of the doctor in healthcare systems	2.0	
CP5 - Practical procedures	2.0	
CP4 - Prescribe drugs	2.0	
CP8 - Legal principles	1.3	Insufficient (0.0 to <1.5)

^a^ 6-point Likert scale (0-5)

The lowest scored items of the questionnaire were found in medical emergencies, practical procedures and legal principles:

- ‘Medical emergencies’ included “trauma care according to guidelines” (1.59), “advanced life support according to guidelines” (1.72) and “treating medical emergencies” (2.04).
- ‘Practical procedures’ showed low scores in “performing blood transfusions“ (0.82), “intravenous therapy and using infusion devices“ (1.47), “urinary catheterization“ (1.50), “basic respiratory tests“ (1.77) and “venous catheterization“ (1.80). The highest scored items are “administering oxygen” (3.04), “urinalysis“ (2.65), “blood pressure measurement” (2.64), “suturing” (2.61) and “move and handle patients” (2.60).
- CP factor ‘Prescribe drugs’ showed low scores in “prescribing in a clear and precise way“ (2.14), “associating medications“ (2.19) and “reviewing the adequacy of drug“ therapy (2.40). The related K factor ‘Drugs and prescription’ included items “drug interactions“ (2.43), “pharmacokinetics and pharmacodynamics“ (2.35), “utilization of blood transfusions and blood products“ (2.10) and “complementary/alternative medicine“ (1.95).
- CP factor ‘Legal principles’ included very poorly scored items such as “certifying deaths“ (1.64), “applying national and European legislation to healthcare“ (1.64) and “requiring autopsies“ (1.06). The equivalent K factor ‘Role in healthcare systems’ included items such as “relevant legislation for medical practice” (2.32), “principles of clinical audit“ (2.26), “knowledge about healthcare systems“ (2.10) and “professional regulation systems“ (1.77).

**Fig. 3:**
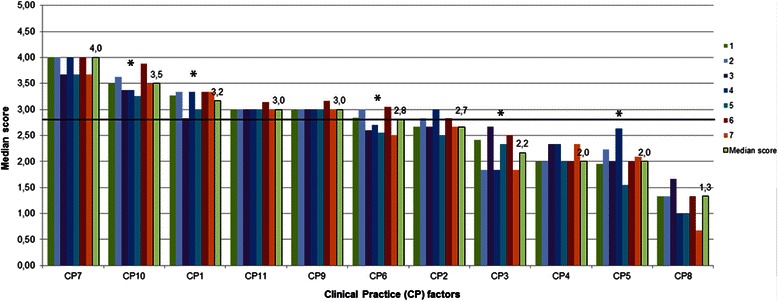
Clinical Practice factor scores per medical school. Random numbers from 1 to 7 were attributed to different schools. The horizontal line shows the median value of Clinical Practice factors (2.8). * significant differences among schools (*p* < 0.05)

**Fig. 4:**
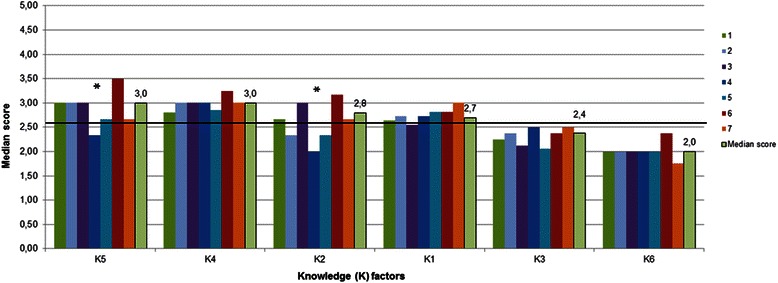
Knowledge factor scores per medical school. Random numbers from 1 to 7 were attributed to different schools. The horizontal line shows the median value of Knowledge factors (2.6). * significant differences among schools (*p* < 0.05)

Factor scores were compared among medical schools. Multiple comparisons highlighted significant differences (*p* < 0.05) in factors CP1, CP3, CP5, CP6, CP8, CP10, K2 and K5. CP7 was the highest and CP8 the lowest scored factor in all medical schools (Figs. 3 and 4).

### Clinical Settings

More than 90 % of graduates experienced having contact with patients in *Clinical Settings* CS2*,* CS3*,* CS4*,* CS5*,* CS6*,* CS8*,* CS9 and CS11*.* Conversely, CS13 (47 %), CS12 (59 %) and CS7 (60 %) showed the lowest percentages (Table 6). Only 24 % of graduates had contact with patients in all 14 *Clinical Settings*.

**Table 6:** Percentage of graduates who experienced having contact with patients in core Clinical Settings (in descending order)

Clinical settings	N (%)
CS2 - Care of general (internal) medical patients in medical admission units	377 (99)
CS4 - Care in the community/family practice/primary care	377 (99)
CS3 - Care of general surgical patients in surgical admission units	375 (98)
CS5 - Care for elderly patients	372 (98)
CS6 - Care for sick children	370 (97)
CS9 - Obstetric and gynaecological care	370 (97)
CS8 - Care for mentally ill patients	365 (96)
CS11 - Care of patients with specialized medical conditions (eg haematology, renal)	346 (91)
CS14 - Care of patients with specialized surgical conditions (eg cardiac surgery, urology)	331 (87)
CS1 - Care of acutely ill patients in Casualty/Accident and Emergency units	297 (78)
CS10 - Care for critically ill patients in Intensive Care Units	273 (71)
CS7 - Care for the dying, palliative care	230 (60)
CS12 - Anaesthetic care	227 (59)
CS13 - Rehabilitation medicine	179 (47)

**Fig. 5:**
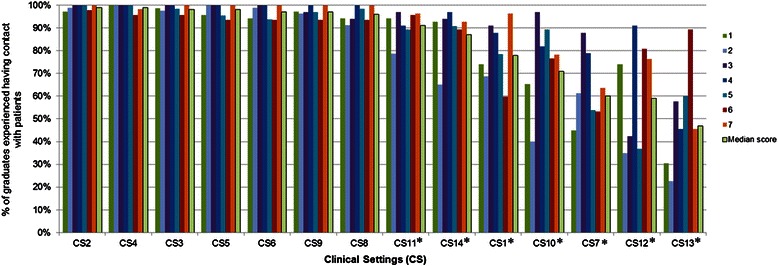
Percentage of graduates who experienced having contact with patients in core Clinical Settings per medical school. * significant differences between schools (*p* < 0.05)

Graduates from different medical schools showed average percentages of contact with patients (considering all 14 *Clinical Settings*) ranging from 75 % (school 2) to 91 % (school 4). Considering specific settings, percentages varied from 23 % (CS13, school 2) to 100 % (several examples in different schools). Multiple comparisons showed significant differences (*p* < 0.05) among graduates from different schools in 7 settings (CS1, CS7, CS10, CS11, CS12*,* CS13, CS14) (Fig. 5).

## Discussion

We have validated a questionnaire that can serve as a SWOT analysis tool for medical education at a national level. In fact, the TEST study provides a pioneer input on Portuguese medical graduates’ self-assessed clinical competence against an European referential. For the first time, the effectiveness of Portuguese medical schools in delivering core competences and exposing students to core clinical settings is explored, and areas where curricula may benefit from improvements are highlighted.

### How did graduates self-assess their acquisition of core competences?

The median values of *Clinical Practice* and *Knowledge* factors correspond to a level of self-assessed competence between sufficient and good. The majority of factors showed a good level of competence. Important differences were found among CP factors, scores ranged from 1.3 (insufficient) to 4.0 (very good) in a 0 to 5 Likert scale. Among K factors, variation was not that evident (from 2.0 to 3.0). Furthermore, CP factors also showed the highest and the lowest scored items in the questionnaire. These results show that the dispersion of results was greater in CP competences.

Important aspects of medical practice were self-assessed above the median CP value in all medical schools: “Consultation with a patient and management plan”, “Ethical principles”, “Psychological and social aspects of illness”, “Evidence-based medicine and scientific principles”, as well as “Population health and healthcare system”. Knowledge on “Public Health” was also scored above the median K value in all schools.

On the other hand, CP factors “Medical emergencies”, “Practical procedures”, “Prescribe drugs” and “Legal principles” scored below the median CP value in all medical schools. These results are consistent with other studies on self-assessed competence in recent graduates or junior doctors [28, 29].

Poor grouped self-assessments in “Medical emergencies” might be explained graduates’ feelings of incapacity to deal with emergent clinical scenarios, fear of making mistakes and limited opportunities for practice in emergency settings during the medical courses. Regarding “Practical procedures”, low self-assessment scores may reflect the same needs of improvement in medical curricula, namely more opportunities for practice in simulated and real patients.

In what refers to the prescription of drugs, *Clinical Practice* (Prescribe drugs) and *Knowledge* (Drugs and prescription) factors were coherent, which suggests internal consistency of the questionnaire and emphasizes low self-assessed competence in this domain. These results may reflect the lack of practical or case-based teaching approaches to therapeutics which could be more adequate for transition to postgraduate training than the frequently undertaken theoretical approaches.

Competence on “Legal principles” in medical practice was very poorly scored by recent graduates. Both the *Clinical Practice* (CP8) and the equivalent *Knowledge* (K6) factor on that domain showed that graduates are not familiar with relevant legislation and medical paperwork/administrative tasks.

All the above-mentioned domains of clinical competence are required of Portuguese medical graduates in order to provide quality patient care, notwithstanding that they are under supervision and integrated in medical teams. The follow-up on recent graduates through the ‘Common Year’ and residency would clarify whether these self-assessed deficits in the development of clinical competence are maintained or improved, provide more information for undergraduate program evaluation and might show latent insufficiencies in clinical competence. This longitudinal approach has been followed in the United Kingdom, where research used quantitative and qualitative methods such as interviews of graduates, their colleagues, senior doctors and other healthcare professionals [28].

### Did graduates experience contact with patients in core clinical settings?

The vast majority of graduates experienced contact with patients in most of the core clinical settings, including internal medicine and surgery admission units, primary care, care of elderly patients, children, pregnant women and psychiatric patients.

Nevertheless, around one quarter of graduates did not have contact with acutely ill patients in emergency or intensive care units, which may link to the above-mentioned results of lower self-assessed competence regarding “Medical emergencies”. This is also consistent with studies on first-year residents [20]. Percentages of graduates who had contact with patients in other settings may also unveil deficiencies in undergraduate education: only 40 % of graduates experienced contact in palliative and anesthetic care. In fact, the item “providing care of the dying and their families” (2.08) was the lowest scored of its factor, which shows consistency in the study’s findings.

*Clinical Settings* with the lowest percentages might be underused for learning purposes during undergraduate education, which may impact on graduates’ preparation and confidence to deal with specific types of patients and healthcare needs.

However, we point out that the questionnaire did not evaluate the quality or quantity of the learning experiences in clinical settings. Further research may show other settings in which learning experiences were not frequent and/or did not have positive educational value, thus contributing to lower clinical competence among medical graduates.

### Are there differences between graduates from different medical schools?

We found that the highest and lowest scored factors were common among cohorts of graduates from different Portuguese medical schools. In fact, no single school or groups of schools showed consistently high or consistently low results across the various parts of this study. Differences among schools were smaller than differences among different clinical competences, knowledge domains and clinical settings. This conclusion was also obtained in previous studies on the effectiveness of medical undergraduate programs in the United Kingdom [28]. This may indicate that the school effect is less important than the effect of high-quality clinical experiences in specific disciplines or active learning behaviours.

Nevertheless, significant differences among medical schools were found in some CP and K factors: school 6 scored the highest in 4 out of 6 K factors. Importantly, differences among schools with regard to percentages of contact with patients in some *Clinical Settings* are sometimes substantial, namely in the settings with the lowest percentages, such as emergency and intensive care units, palliative care, anesthetic care, rehabilitation medicine and specialized surgical and medical conditions: for example, while 89.4 % of graduates from school 7 had contact with patients in rehabilitation units, only 22.5 % of graduates from school 3 had the same learning opportunities (in fact, school 3 showed the lowest percentages in 4 out of 14 *Clinical Settings*).

Differences among schools may be explained by an analysis of differences in their medical curricula, teaching-learning strategies or even assessment methods. Regarding *Clinical Settings*, differences may reveal that only some medical schools have acknowledged the importance of all the Tuning core clinical settings in undergraduate education. Marked differences among schools can also be influenced by the available healthcare units and the collaboration with teaching hospitals.

We found that Portuguese medical schools may not be considerably different with regard to their effectiveness in delivering core competences, but this requires further research. In fact, comparative research may lead to substantial progress in medical education [30]. Outcome-based program evaluations might stimulate faculty development, guide recently established medical schools [21] and strengthen schools’ accountability as elements of larger healthcare systems. Medical schools’ collaborative efforts for program evaluation and detection of areas needing improvement in undergraduate education have been developed, which included recent graduates’ self-assessments [28].

### Limitations

The TEST study analyzed recent medical graduates’ grouped self-assessment of core competences in order to infer about real clinical competence and consequently about the effectiveness of undergraduate programs. This emphasizes the need to interpret results with care, considering beforehand some relevant topics.

We consider that our sample size is representative of the study population. In fact, almost one out of four Portuguese medical graduates answered the survey - which fulfilled our aim of five participants per item for the purpose of factor analysis - and the sample closely resembles the population in terms of gender, age and admission contingent. Also, medical schools are represented in the sample in accordance to their number of admissions, and cohorts of graduates from different schools were not different in terms of gender, age or modality of admission.

Questionnaires were implemented in preparation lectures for the national exam to access residency, which might have induced a selection bias that favored more interested students or, conversely, students that need improvement. Moreover, the possibility of more than one response to the survey per person needs to be considered, since paper and electronic versions of the questionnaire were distributed. However, explicit indications were given so that only one version of the questionnaire was filled and we have no reasons to believe that a selection bias had a significant impact in the study.

The final year of the medical course (which graduates had completed in July) might have had considerable impact on graduates’ self-assessments. Hence, the study’s findings may be more reflective of the later stages of medical curricula, especially clinical experiences in the final year. This suggests that the lowest scored factors highlight areas of clinical competence and knowledge that might be improved by the educational development of the transition period between undergraduate and postgraduate training. In fact, the final year of the medical course in Portugal shows some of the problems pointed out in the literature [31].

Regarding the study instrument, Tuning core outcomes are reassuring in terms of content validity and the exploratory factor analysis yielded meaningful factors that explained a great proportion of the variance of answers. Nevertheless, concerns regarding the questionnaire’s face validity may be raised. We used a 6-point Likert scale of levels of competence (from non-existent to excellent competence) in which graduates had to define what “sufficient” competence meant, for each item of questionnaire. The concept of “sufficient competence” may be difficult to define and interpreted differently among graduates. Also, they may have different interpretations of the concept depending on their medical school, since learning outcomes, curricula, learning and assessment experiences might have influenced their expectations and standards. These limitations may harm the validity of comparisons among schools. A non-differential bias may also explain why only one of the factors (CP8) was self-assessed below a sufficient level of competence; in order to obtain a clearer view on the highest and lowest areas of self-assessed competence, we interpreted factor scores considering their absolute scores and their position in relation to the median value of all *Clinical Practice* or *Knowledge* factors. Narrative descriptions might improve the questionnaire’s face validity by associating each level of competence in each item to specific clinical scenarios.

Importantly, competence on practical procedures seems to be more accurately self-assessed than knowledge [32]. Also, the focus on medical knowledge is reduced at later stages of the medical courses in Portugal. Graduates may therefore have more difficulties to self-assess their knowledge than their competence in procedural skills, which leads us to consider that this study’s findings regarding *Clinical Practice* factors show a better correlation with Portuguese graduates’ real competence and prospective difficulties in the ‘Common Year’.

Grouped self-assessments are particularly important for program evaluation in the Portuguese context, where it is difficult to define an objective measure of clinical competence which can be considered the gold-standard at a national level. Also, research based on grouped self-assessments is inexpensive and stimulates graduate’s self-reflection and engagement in medical education. In fact, the purpose of this study is not to obtain a precise measurement of individual or even group competence, but to provide important data for the purpose of evaluating program effectiveness and driving outcome-based curricular improvement. We believe that results regarding core clinical competences with the lowest self-assessed scores are the most relevant for program evaluation purposes and deserve more attention from medical schools. Further research may refine this pilot initiative, emphasizing domains of clinical competence which are more prone to deliver valid grouped self-assessment data, and improving the survey’s implementation in collaboration with all Portuguese medical schools.

## Conclusion

The TEST study developed a valid and sensitive questionnaire that supports national SWOT analysis on the acquisition of core competences in undergraduate medical education. In fact, graduates’ self-assessments highlighted deficits in core clinical competences at a national level. Results suggest that Portuguese graduates are not fully prepared for clinical practice according to the Tuning Project’s European referential. Medical emergencies, practical procedures, prescribe drugs and legal principles in medical practice showed the lowest self-assessment scores. Graduates had the least contact with patients in the emergency rooms, intensive care units, palliative, rehabilitation and anesthetic care. Cohorts of graduates are similar among medical schools, revealing mostly the same stronger and weaker domains of self-assessed competence and knowledge. Curricular improvements in the above-mentioned areas and the educational development of the transition period between undergraduate and postgraduate education ought to be considered. The TEST study supports the idea that outcome-based program evaluations, relying on graduates’ grouped self-assessments, can contribute to inform changes in medical education at a national level.

## Abbreviations

ECS-UM: Escola de Ciências da Saúde da Universidade do Minho (School of Health Sciences - University of Minho, Braga)
EFA: Exploratory factor analysis
FCS-UBI: Faculdade de Ciências da Saúde da Universidade da Beira Interior (Faculty of Health Sciences - University of Beira Interior, Covilhã)
FMUC: Faculdade de Medicina da Universidade de Coimbra (Faculty of Medicine – University of Coimbra)
FMUL: Faculdade de Medicina da Universidade de Lisboa (Faculty of Medicine - University of Lisbon)
FMUP: Faculdade de Medicina da Universidade do Porto (Faculty of Medicine - Unversity of Porto)
ICBAS: Instituto de Ciências Biomédicas Abel Salazar (Institute of Biomedical Sciences Abel Salazar - University of Porto)
KMO: Kaiser-Meyer-Olkin
NMS/FCM: NOVA Medical School
OBE: Outcome-based education
SPSS: Statistical Package for Social Sciences
SWOT: Strengths, Weaknesses, Opportunities and Threats.
